# Burn‐induced myocardial depression in a pediatric patient leading to fulminant cardiogenic shock and multiorgan failure requiring extracorporeal life support

**DOI:** 10.1002/ccr3.2667

**Published:** 2020-02-22

**Authors:** Apurva Panchal, Joseph Casadonte

**Affiliations:** ^1^ Department of Pediatrics University of Kansas Health System Kansas City KS USA; ^2^ Department of Pediatrics Dell Children’s Medical Center of Central Texas Austin TX USA

**Keywords:** beta‐blockers, burn‐induced cardiogenic shock, extracorporeal life support, multiorgan failure, pediatric burn, renal replacement therapy

## Abstract

Cardiac stress is a critical determinant of outcomes associated with severe thermal injury. The cardiovascular response to a catecholamine‐mediated surge from severe burns passes through two phases. Initial hypovolemia with myocardial depression leads to a low cardiac output, which then progresses to a hyperdynamic‐hypermetabolic phase with increased cardiac output.

## Graphical abstract

Physicians taking care of pediatric burn patients should be familiar with the concept of judicious fluid resuscitation and careful use of beta‐blocker in severe burn with concern of myocardial depression.

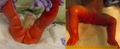

## 1 INTRODUCTION

Cardiac stress is a critical determinant of outcomes associated with severe thermal injury. The cardiovascular response to a catecholamine‐mediated surge from severe burns passes through two phases. Initial hypovolemia with myocardial depression leads to a low cardiac output, which then progresses to a hyperdynamic‐hypermetabolic phase with increased cardiac output.

Severe burn, which is defined as burn involving more than 40% of total body surface area (TBSA), has profound and prolonged effects on the cardiovascular system.^1^, ^2^, ^3^ The cardiovascular changes will dictate morbidity and mortality associated with the burn. The magnitude of these changes is proportional to the severity of the burn.^1^, ^2^, ^4^ Immediately after the burn injury, cardiac output may get compromised, secondary to acute myocardial dysfunction and increased vascular permeability. Significant intravascular hypovolemia from the burn injury exaggerates low cardiac output.^1^ These acute changes may last for a few days before evolving into hyperdynamic state of increased cardiac output and myocardial oxygen demand. The latter state has been reported to persist for a few years.^2^

The burn‐induced hyperdynamic‐hypermetabolic state has been well studied. However, there only handful of case reports describing the acute cardiovascular changes secondary to burn. We report a case of a burn‐induced fulminant myocarditis in a 14‐month‐old girl who developed cardiogenic shock and required extracorporeal membrane oxygenation and renal replacement therapy for survival.

## 2 CASE PRESENTATION

**Figure 1:**
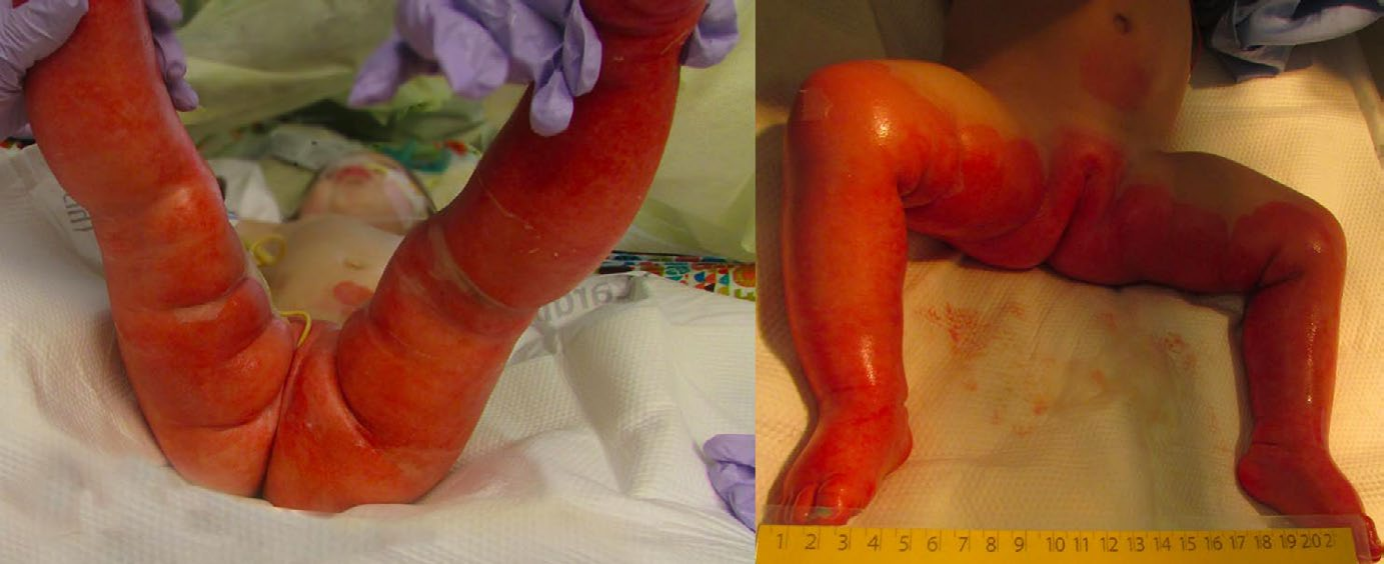
Partial to full‐thickness scald burn involving more than 20% TBSA on presentation

A 14‐month‐old girl was admitted to our pediatric intensive care unit (PICU) at the University of Kansas Health System with scald burn from hot water in a bathtub. Reportedly, her father left her in the tub for few seconds to check on her sibling. On initial assessment, there were deep partial to full‐thickness burn wounds on the lower abdomen, perineum, bilateral buttocks, and lower extremities. Around 28% of the total body surface area (TBSA) was affected (Figure 1). Her vitals were age appropriate except for tachycardia with an initial heart rate in the 180 s per minute. Rest of the physical examination was reassuring. She had a significant past medical history of multiple rib fractures of unknown etiology at eight months of age. Child protective services were involved at that time.

Upon admission to the PICU, the resuscitation fluid was calculated as per the modified Parkland formula (4 mL/kg/%TBSA of burn). Along with the maintenance fluid, the resuscitation fluid (normal saline) was administered over the next 24 hours. The fluid rate was titrated based on her hourly urine output (UOP). She received additional crystalloid fluid boluses to achieve a goal UOP of 1‐2 mL/kg/h. Her heart rate stayed high in the 150‐200 beats per minute range over the first 48 hours despite aggressive fluid resuscitation. Because of concern of the hypermetabolic syndrome contributing to her tachycardia, she was started on 2 mg of propranolol three times a day on the second day of hospitalization. Propranolol dose was increased to 2.5 mg three times a day subsequently.

On the hospital day 4, she developed fever, abdominal distension, respiratory distress with retractions, and decreased UOP. Blood culture, urine culture, and chest X‐ray were obtained. Empiric antibiotics were initiated with a concern of sepsis. She was placed on high‐flow nasal cannula oxygen support with 100% fractionated oxygen flowing at 8 L per minute. Her chest X‐ray was negative for pulmonary edema, atelectasis, pneumonia, and any other acute changes. Her condition improved for a brief period prior to decompensation. Additional fluid boluses had little to no effect on her UOP. Abdominal ultrasound ruled out ascites and secondary abdominal compartment syndrome. Her breathing continued declining, with more retraction and tachypnea. Her cardiovascular system worsened, with more tachycardia and new onset of hypotension. Signs of poor end‐organ perfusion were evidenced with prolonged capillary refill, high lactic acidosis (9.6 mmol/L), and low regional (cerebral and renal) venous oxygen saturation of 40%‐50%, measured by near‐infrared spectroscopy (NIRS).

Her respiratory support was escalated from a high‐flow nasal cannula to invasive positive pressure ventilation. After proper premedication with ketamine, versed, and vecuronium, she was intubated with a 4.0 cuffed endotracheal tube successfully. Central venous line was placed in her right internal jugular vein to obtain central venous pressure (CVP) for assessing the intravascular volume status. Her initial CVP was 4 cm H_2_O. Epinephrine infusion was initiated at 0.1 mcg/kg/min and titrated up to achieve mean arterial blood pressure of 50 mm Hg or higher and to improve other parameters of perfusion. A repeat chest X‐ray showed bilateral pulmonary edema, and her latest B‐type natriuretic peptide was more than 5000 pg/mL. Due to minimal effect of high dose epinephrine on her cardiovascular system, an echocardiogram was obtained emergently and showed depressed cardiac function with an ejection fraction of 40% and shortening fraction of 21%.

Over the course of the night, her condition continued deteriorating with rising lactate, low venous oxygen saturations on NIRS, and anuria. She remained tachycardic and hypotensive despite aggressive vasoactive medications support and discontinuation of propranolol. At this point, the plan was made to initiate extracorporeal life support (ECLS) for cardiac failure as well as renal replacement therapy (RRT) for her acute renal failure. Her all blood and urine cultures were reported negative subsequently.

## 3 DISCUSSION

Classically, the cardiovascular changes from a burn follow two phases. The first phase, described as the "ebbed" phase, starts immediately after a burn injury and can last from 2 to 3 days.^1^ Cardiac stress is the hallmark of the ebbed phase. Multiple factors, including catecholamine‐induced (stress‐induced) cardiomyopathy, high serum level of vasopressin, angiotensin II and neuropeptide Y, and the release of certain cytokines and gut‐derived factors have been postulated to instigate myocardial depression during this phase.^1^, ^3^, ^4^ Profound inflammation from the severe burn with the release of proinflammatory cytokines like tissue necrosis factor alpha and macrophage inhibitory factor, which are known to be cardiac depressants, is a key feature of the ebbed phase.^1^, ^3^ Evidence also suggested a direct myocardial cell injury and alteration in myocardial intracellular calcium homeostasis contribute to myocardial depression.^4^ In addition to myocardial depression, burn‐induced inflammation impairs biventricular diastolic compliance and increases vascular permeability.^4^ Massive intravascular volume loss from the burn itself, along with myocardial dysfunction and capillary leak, leads to low cardiac output and subsequent hypoperfusion and impaired microcirculation.^4^ Compromised circulation causes poor wound healing, burn zone extension, and infection.^2^, ^4^ Studies have shown that hypoperfusion and infection are independent risk factors for multiple organ dysfunction and death in burn patients.^5^ The chances of worst outcomes are high in pediatric population.^4^

The first phase is then progressed to the phase of hypermetabolism, also known as "flow" phase.^1^ Unlike ebbed phase, the flow phase is characterized by the increased cardiac output (provided that adequate resuscitation was done during the first phase), increased myocardial oxygen consumption and hypermetabolism.^1^, ^2^ This phase is facilitated not only by catecholamine but also by corticosteroids and glucagon. Hypermetabolism from burn has a long‐lasting effect on the cardiovascular system, and it can last up to 2‐3 years, postburn injury.^2^, ^6^ Description of the flow phase along with the detailed management guidelines, including anabolic steroid, beta‐blockers, and insulin, to limit the hypermetabolism and related complications during this phase has been provided in various studies.^6^

On the other hand, few animal studies and case reports have provided the insight of cardiovascular changes during the ebb phase.^1^, ^7^, ^8^ Most of them lack information on the effective screening and management of cardiovascular injury during the ebb phase. Guidelines are not available to screen patients at risk for acute cardiovascular deterioration in the acute phase of burn injury. Fluid resuscitation to restore intravascular volume and preload in the early management of burn has not been very effective in restoring the cardiovascular system in many cases.^8^ Ongoing fluid resuscitation to achieve goal UOP in the phase of low cardiac output secondary to myocardial depression may worsen the hemodynamic status further, and it can be hazardous.^9^ Fluid resuscitation without continuous assessment of cardiovascular functions could lead to fluid overload also known as fluid creep. Fluid creep has been identified as an independent risk factor for myocardial depression and subsequent high morbidity and mortality in burn patients.

Our patient suffered from a multifactorial myocardial dysfunction and cardiogenic shock due to burn‐induced profound catecholamine surge, massive inflammation, and sepsis. Despite aggressive vasoactive support, her progressive myocardial depression led to multiorgan failure. We also fear that the early use of beta‐blocker in our patient might have hindered the effect of vasoactive agents. Although, the beneficial effect of beta‐blocker in thermal injury has been proven,^10^ the timing to initiate the intervention remains controversial since blocking beta receptors may further reduce cardiac functions in immediate postburn period.^11^ She required ECLS for cardiovascular support and RRT for her acute renal failure. She suffered from a massive hemorrhagic stroke while being on ECLS. After 5 months of extensive hospital course, she was discharged home with significant neurological morbidity and G‐tube dependency.

## 4 CONCLUSION

Cardiovascular stress is common after thermal injury, and it determines the outcome associated with thermal injury. Although infection is the most common cause of fulminant myocardial depression in pediatric patients, this case illustrates that the burn‐induced myocardial insult may be a risk factor for fulminant myocardial depression and a secondary cardiogenic shock. We recommend screening for myocardial insult by routine serial echocardiograms in pediatric patients with severe burn involving more than 20% of TBSA. We also suggest delaying the use of beta‐blocker for hypermetabolism and judicious fluid resuscitation until the ventricular function can be determined.

## CONFLICT OF INTEREST

Authors declared no conflicts of interest.

## AUTHOR CONTRIBUTIONS

AP and JC: participated in the management of this patient as well as in the preparation and edition of this manuscript. Both authors: read and approved the final manuscript.

## ETHICS APPROVAL AND CONSENT TO PARTICIPATE

Informed consent was taken from the parents of child prior to start of study and taking of photographs. The parents were also made aware that information and pictures taken were for academic purpose and publication.
